# Age-dependent yield of screening for undetected atrial fibrillation in stroke patients: the Find-AF study

**DOI:** 10.1007/s00415-013-6935-x

**Published:** 2013-05-01

**Authors:** Rolf Wachter, Mark Weber-Krüger, Joachim Seegers, Frank Edelmann, Janin Wohlfahrt, Katrin Wasser, Götz Gelbrich, Gerd Hasenfuß, Raoul Stahrenberg, Jan Liman, Klaus Gröschel

**Affiliations:** 1 Clinic for Cardiology and Pneumology, DZHK (German Center for Cardiovascular Research), University of Göttingen, Göttingen, Germany; 2 Clinic for Neurology, University of Göttingen, Göttingen, Germany; 3 Institute of Clinical Epidemiology and Biometry, University of Würzburg, Würzburg, Germany; 4 Department of Neurology, University of Mainz, Mainz, Germany; 5 Department of Cardiology and Pneumology, Universitätsmedizin Göttingen, 37075 Göttingen, Germany

**Keywords:** Age, Paroxysmal atrial fibrillation, Screening, Stroke, Atrial fibrillation, Cohort study

## Abstract

Diagnosis of paroxysmal atrial fibrillation (AF) in stroke patients is challenging, but highly clinically relevant. The percentage of stroke patients with permanent AF increases with age, but limited data are available for the age-dependent yield of paroxysmal AF by Holter monitoring. Patients with acute cerebral ischemia were included into the prospective observational Find-AF study. Patients free from AF at presentation received 7 day Holter monitoring. We calculated the percentage of otherwise undetected paroxysmal AF and the number needed to screen for age groups under 60 years, and in 5 year clusters from the age of 60 up to 85 and older. 272 patients were included, 43 (15.8 %) had AF at admission, 33 patients with paroxysmal AF were identified by 7 day Holter (*n* = 29) or medical history (*n* = 4).The yield of 7 day Holter ECG clearly increased with older age (*p* = 0.004): <60 years: 5 %, 60–64 years: 5 %, 65–69 years: 7 %, 70–74 years: 11 %, 75–79 years: 13 %, 80–84 years: 25 %, ≥85 years: 39 %. The number needed to screen (NNS) to find one patient with paroxysmal AF decreased with age: ≤60 years: 18, 60–64 years: 20, 65–69 years: 14, 70–74 years: 9, 75–79 years: 8, 80–84 years: 4, ≥85 years: 3, respectively. In patients <65 years, all AF cases were detected by Holter ECG. The percentage of paroxysmal AF in stroke patients increases with age. The 7 day Holter ECG is most efficient in elderly patients.

## Introduction

Atrial fibrillation (AF) is a frequent cause of ischemic stroke and patients with atrial fibrillation bear an increased risk of stroke recurrence [1, 2].

The yield of AF detection clearly increases by prolonging the monitoring interval: In our trial, 7 day Holter monitoring identified significantly more AF cases than shorter monitoring intervals [3] and these results are in line with those of several other trials [4]. Patients with detected AF show substantial alterations in left atrial size and function [5] as well as neurohumoral activation [6]. However, detailed data on the yield of this diagnostic procedure in relation to age are not available thus far. Hence, the aim of this study was to evaluate the detection rates of paroxysmal AF using 7 day Holter monitoring in relation to the patients’ age.

## Materials and methods

### Patients and AF detection

Find-AF is a single centre prospective observational trial (ISRCTN 46104198). Patients presenting at the university hospital of Göttingen, Germany, with signs of cerebral ischemia were included. Those without AF on baseline ECG received 7 day Holter monitoring. The recordings were analysed by specially trained personal and reviewed by a specialist in cardiac electrophysiology. A clinical follow-up visit took place after one year. Further details on the study outline have been published previously [3]. The study was approved by the local ethic’s committee prior to inclusion of the first patient. This study complies with the Declaration of Helsinki and all patients gave written informed consent.

### Definitions and statistical analyses

Continuous data are given as mean ± standard deviation, categorical variables are given as absolute numbers (percent), unless otherwise stated. Groups were compared by ANOVA (followed by Tukey’s post hoc test), Mann–Whitney *U* test, Chi square test or Fisher’s exact test, as appropriate. Statistical tests were performed with SPSS Statistics 20.0.0 (IBM, Chicago, Illinois, USA).

## Results

### Study population

Two hundred and eighty one consecutive patients were included, of whom eight had a final diagnosis other than cerebral ischemia (one of these eight patients presented with AF) and were excluded from further analysis. One patient withdrew consent. 43 (15.8 %) of the remaining 272 patients had AF at presentation, the other 229 patients received 7 day Holter ECG. Paroxysmal AF was detected in 29 patients (12.6 %), an additional four patients had a history of paroxysmal AF, but no AF on 7 day Holter ECG. Clinical characteristics of the complete cohort have previously been reported [3].

Table 1 shows baseline characteristics (AF on admission, *n* = 43; paroxysmal AF *n* = 33; patients without AF, *n* = 196) of the study participants.

**Table 1:** Study participant characteristics

	No atrial fibrillation (*n* = 196)	Paroxysmal atrial fibrillation (*n* = 33)	Atrial fibrillation on admission (*n* = 43)	*P* value No AF vs. paroxysmal AF	*P* value AF on admission vs. paroxysmal AF
Age	67 ± 13	76 ± 11	79 ± 6	**0.001**	0.401
Female gender	82 (41.8 %)	14 (42.4 %)	25 (58.1 %)	1.000	0.247
BMI	27.6 ± 5.9	27.6 ± 5.8	28.1 ± 5.3	0.998	0.932
NIH-SS	2 (1;4)	5 (4;9)	4 (3;8)	**<0.001**	0.611
Modified Rankin Scale	2 (1;3)	3 (2;4)	3 (2;4)	**0.021**	0.502
Stroke severity^a^				0.002	0.112
TIA	69 (35.2 %)	3 (9.1 %)	13 (30.2 %)		
Minor stroke	54 (27.6 %)	8 (24.2 %)	7 (16.3 %)		
Major stroke	73 (37.2 %)	22 (66.7 %)	23 (53.5 %)		
TOAST classification				**<0.001**	**0.001**
Large artery atherosclerosis	39 (19.9 %)	0 (0.0 %)	0 (0.0 %)		
Cardioembolic	28 (14.3 %)	15 (45.5 %)	36 (83.7 %)		
Lacunar/small vessels	27 (13.8 %)	0 (0.0 %)	0 (0.0 %)		
Rare/other causes	5 (2.6 %)	0 (0.0 %)	0 (0.0 %)		
Undetermined/multiple Causes	97 (49.5 %)	18 (54.5 %)	7 (16.3 %)		
Heart rate (bpm)	72 ± 13	71 ± 16	80 ± 19	0.894	**0.013**
Systolic blood pressure (mmHg)	144 ± 25	153 ± 25	142 ± 25	0.129	0.161
Diastolic blood pressure (mmHg)	79 ± 13	79 ± 13	84 ± 16	1.000	0.292
Creatinine (mg/dl)	1.0 ± 0.5	1.0 ± 0.5	1.3 ± 0.7	0.921	**0.004**
Haemoglobin (mg/dl)	13.9 ± 1.7	13.6 ± 2.0	12.9 ± 1.8	0.581	0.172
Left atrial diameter (mm)	41 ± 7	44 ± 6	51 ± 8	**0.031**	**0.001**
Left ventricular ejection fraction (%)	62 ± 12	60 ± 10	58 ± 12	0.587	0.824
History of stroke	32 (16.3 %)	5 (15.2 %)	5 (11.6 %)	1.000	0.739
History of TIA	20 (10.2 %)	2 (6.1 %)	4 (9.3 %)	0.749	0.692
Heart failure	10 (5.1 %)	2 (6.1 %)	7 (16.3 %)	0.685	0.284
Hypertension	139 (70.9 %)	28 (84.8 %)	34 (79.1 %)	0.137	0.566
Diabetes mellitus	44 (22.4 %)	7 (21.2 %)	16 (37.2 %)	1.000	0.207
Current Smoker	51 (26.0 %)	4 (12.1 %)	2 (4.7 %)		
Hyperlipidaemia	63 (32.1 %)	17 (51.5 %)	13 (30.2 %)	**0.047**	0.097
Coronary artery disease	22 (11.2 %)	12 (36.4 %)	9 (20.9 %)	**0.001**	0.196
Peripheral artery disease	5 (2.6 %)	2 (6.1 %)	1 (2.3 %)	0.266	0.576

Bold values indicate statistical significance at *p* < 0.05

^a^Minor stroke: symptoms resolved completely within 30 days or NIH stroke scale changed by ≤3 points; Major stroke: neurologic deficit persisted after 30 days or NIH stroke scale score increased by >3 points

As a first finding, baseline characteristics as well as stroke severity were similar in patients with paroxysmal AF as compared to patients with AF on admission. Patients with AF (either paroxysmal or on admission ECG) were significantly older, they had more severe and more disabling strokes than patients with SR (Table 1).

### Age-dependent detection of atrial fibrillation

**Fig. 1:**
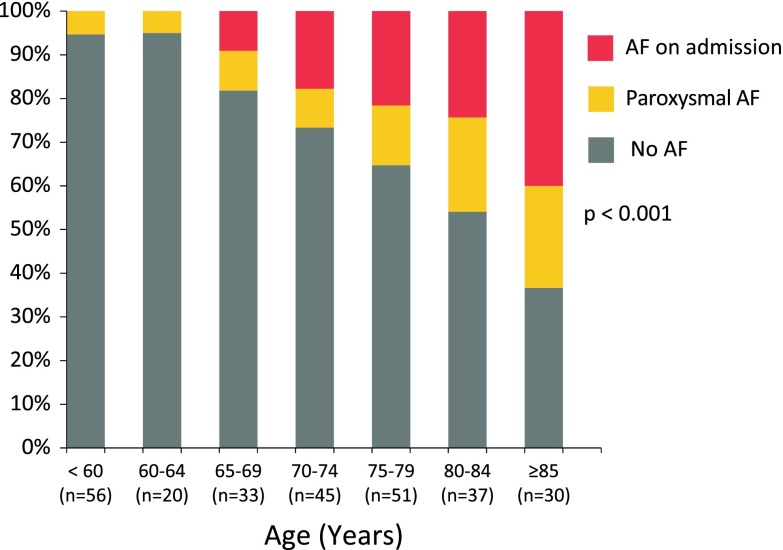
Cumulative percentage of study participants with no AF (*grey*), paroxysmal AF (detected by 7 day Holter or medical history, *orange*) and AF on admission ECG (*red*) in relation to participants’ age

We formed age clusters and found a clear increase of the AF rate with age (*p* < 0.001). This finding was consistent for paroxysmal AF as well as for AF on admission ECG (see Fig. 1). In stroke patients younger than 65 years, all AF cases were detected by Holter ECG. In patients older than 65 years, approximately 1/3 of AF patients had paroxysmal AF.

**Fig. 2:**
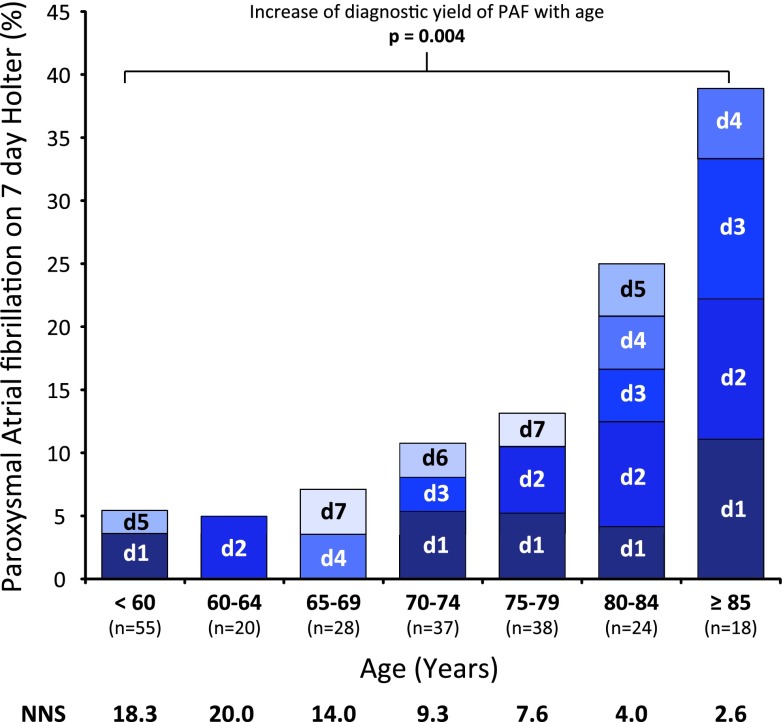
Percentage of study participants with (previously undiagnosed) paroxysmal AF in relation to age. Only participants with no history of paroxysmal AF and an evaluable 7 day Holter ECG were included in this analysis (*n* = 220). *NNS* number needed to screen to detect one patient with paroxysmal AF by 7 day Holter ECG within age groups

Figure 2 shows the 7 day Holter ECG results for all patients with evaluable Holter ECGs and without a history of paroxysmal AF (*n* = 220) as well as the day of first AF documentation. The diagnostic yield of 7 day Holter ECG increased with age (*p* = 0.004), while the number needed to screen to detect one case of paroxysmal AF decreased.

## Discussion

Our study has three major findings:

1. Patients with paroxysmal AF (newly detected by 7 day Holter ECG or previously known) show clinical characteristics and stroke features similar to patients with permanent AF.
2. The prevalence of paroxysmal AF clearly increases with age, while the number needed to screen decreases.
3. All AF cases detected in younger patients were detected by means of prolonged ECG monitoring.

Patients with known AF have more severe strokes [7], however, previously this was not shown for patients with paroxysmal AF diagnosed by 7 day Holter ECG. This finding may be regarded as an indirect hint that patients with paroxysmal AF >30 s (definition used in this study according to current guidelines) represent a relevant stroke subpopulation with equally severely disabling strokes.

It is well established that the risk of stroke in paroxysmal and permanent AF is similar and that both groups equally benefit from primary stroke prevention by means of oral anticoagulation [7, 8]. However, whether patients identified by Holter ECG benefit similarly from secondary prevention therapy (especially anticoagulation) still remains to be determined [9], but is of major interest for further research.

Our data are in line with the well-established increase of AF prevalence with age. However, most available data are based on standard 12-lead ECGs. This work extends these findings to a large stroke population with paroxysmal AF detected by extended Holter monitoring.

The potential role of short AF episodes only to be diagnosed with intensified cardiac monitoring in causing stroke and systemic embolism has recently been re-assured by the ASSERT trial, which found that even short episodes of atrial fibrillation (>6 min within 3 months) detected by long-term evaluation of pacemaker devices with atrial leads increase the risk of subsequent stroke by 2.5 [10].

All AF episodes in younger patients within our trial were detected by means of extended Holter-ECG, no patient <65 presented with AF at baseline or had known paroxysmal atrial fibrillation. Considering the fact that AF related strokes show increased stroke severity and young patients have more quality-adjusted life years at stake, this subgroup could also significantly benefit from prolonged monitoring, seeing this method might be their only chance of identifying AF at all.

Some limitations of our study should be considered. Find-AF was a non-controlled single centre trial. Only 69 % of patients had evaluable Holter ECG material >5 days and five Holter ECGs were not evaluable; hence, the prevalence of paroxysmal AF may be underestimated [3]. Other methods, e. g., even more prolonged spells of Holter monitoring or (implantable) loop recorders, may have yielded more AF cases, these methods are currently under investigation [11, 12]. There were only few young patients with cerebral ischemia and paroxysmal AF in our population, so that conclusions regarding this patient group should be re-evaluated in a larger population.

## Summary

The prevalence of paroxysmal AF in stroke patients increases with age. Screening by prolonged Holter ECG is most efficient in older patients.
